# An Exercise-Only Intervention in Obese Fathers Restores Glucose and Insulin Regulation in Conjunction with the Rescue of Pancreatic Islet Cell Morphology and MicroRNA Expression in Male Offspring

**DOI:** 10.3390/nu9020122

**Published:** 2017-02-09

**Authors:** Nicole O. McPherson, Michelle Lane, Lauren Sandeman, Julie A. Owens, Tod Fullston

**Affiliations:** 1 Discipline of Obstetrics and Gynaecology, School of Paediatrics and Reproductive Health, University of Adelaide, Adelaide, South Australia 5005, Australia; michelle.lane@adelaide.edu.au (M.L.); laren.sandeman@adelaide.edu.au (L.S.); julie.owens@adelaide.edu.au (J.A.O.); tod.fullston@adelaide.edu.au (T.F.); 2 Freemasons Centre for Men’s Health, University of Adelaide, Adelaide, South Australia 5005, Australia; 3 Monash IVF Group, Dulwich, South Australia 5056, Australia

**Keywords:** sperm, fertility, nutrition

## Abstract

Paternal obesity programs metabolic syndrome in offspring. Low-impact exercise in obese males improves the metabolic health of female offspring, however whether this occurred in male offspring remained unknown. C57BL/6NHsd (Harlan) mice were fed a control diet (CD; 6% fat, *n* = 7) or a high-fat diet (HFD; 21% fat, *n* = 16) for 18 weeks. After 9 weeks, HFD-fed mice either remained sedentary (HH, *n* = 8) or undertook low–moderate exercise (HE, *n* = 8) for another 9 weeks. Male offspring were assessed for glucose/insulin tolerance, body composition, plasma lipids, pancreatic islet cell morphology and microRNA expression. Founder HH induced glucose intolerance, insulin insensitivity, and hyperlipidaemia in male offspring (*p* < 0.05). Metabolic health was fully restored in male offspring by founder exercise to control levels. Founder HH reduced pancreatic β-cell area and islet cell size in male offspring, and altered the expression of 13 pancreatic microRNAs (*p* < 0.05). Founder HE led to partial restoration of pancreatic islet cell morphology and the expression of two pancreatic microRNAs (let7d-5p, 194-5p) in male offspring. Founder HE reduced male offspring adiposity, increased muscle mass, reduced plasma free fatty acids (FFAs), and further altered pancreatic microRNAs (35 vs. HH; 32 vs. CD) (*p* < 0.05). Low-impact exercise in obese fathers prior to conception, without dietary change, may be a viable intervention strategy to reduce the ill-effects of obesity-induced paternal programming in male offspring.

## 1. Introduction

Paternal obesity now occurs in 28% and 33% of expectant fathers in Australia [1] and the USA [2], respectively. While it has become widely accepted that increasing maternal body mass index (BMI) and poor nutrition during gestation or lactation are associated with obesity and metabolic disorders in children [3,4], there is now a growing body of evidence that a father’s BMI and nutritional intake at conception can also influence the BMI of children [5]. However, human datasets are usually confounded by the inability to delineate whether this paternal influence is due to the common “obesogenic” raising environment or genetic predispositions shared by both father and child. Thus the most direct evidence of programming of offspring’s health via the paternal lineage has been generated by rodent models of obesity (high-fat diet feeding), where environmental versus genetic/epigenetic influences are minimised.

To date, several rodent models of paternal obesity have shown a clear programming effect on offspring phenotypes. Using obesity models in both mice and rat demonstrated that the consumption of a diet high in saturated fat induced obesity and altered metabolic profiles in the males themselves prior to mating. Subsequently, paternal high-fat diet changed both neonatal weights and adult growth trajectories in offspring, manifesting as excess adiposity, glucose and insulin intolerance, and sub-fertility in both male and female offspring, with some effects extending into two generations [6,7,8,9,10,11]. The mechanism for this transmission from the paternal lineage is likely to be epigenetic of nature, with changes to sperm methylation [7,11], sperm small non-coding RNA content [11,12,13,14,15], and global acetylation levels in testes [16] known to be perturbed in obese rodent males. In addition, changes to methylation and the transcription of associated genes have also been reported in offspring adipose and pancreatic tissue [8,9], suggesting that the epigenetic changes in sperm have persistent effects on the embryo and subsequent adult offspring.

While there is now a clear transgenerational influence on offspring health from paternal obesity, the question is raised as to whether these offspring effects are reversible. Diet and exercise as obesity interventions in fathers prior to conception improve metabolic health and present as one of the most logical and cost-effective ways of potentially restoring health in offspring. We have previously demonstrated that diet and/or exercise interventions in high-fat diet-fed obese male mice reduces adiposity, improves metabolic profile and sperm function, and reverses adverse effects on early embryo/foetal development [17,18,19]. However, when we assessed adult female offspring metabolic health, somewhat surprisingly; the greatest health benefits (adipocyte size and restored insulin regulation) resulted from offspring whose fathers underwent a light–moderate exercise intervention, whilst continuing with the high-fat diet feeding regime [12]. This suggested that the positive effects of exercise outweighed the negative effects of increased adiposity and high-fat diet consumption. Furthermore, it implies that weight loss may not be necessary in the father himself to improve the health prospects of offspring from the paternal line. Therefore, we aimed to determine if this same light to moderate exercise regime, without a change to diet in obese males, would also be enough to restore the metabolic health of their male offspring.

## 2. Methods

### 2.1. Founder Animals and Diet

Five-week-old male C57BL/6NHsd (Harlan) mice (*n* = 24) were randomly assigned to one of two diets for an initial feeding period of 9 weeks: (1) control diet (CD) (*n* = 8) (SF04-057; Specialty Feeds, Perth, Australia); or (2) a high-fat diet (HFD) (*n* = 16) which contained 21% fat and was nutrient matched (SF00-219; Specialty Feeds, Perth, Australia). The high-fat diet used in this study has been previously shown to increase adiposity [16,20,21,22]. After the initial feeding period, males fed the HFD were then allocated to one of the following treatments for a further period of 9 weeks: (1) continuation of a HFD (HH) (*n* = 8) with no exercise (sedentary); or (2) HFD with exercise intervention (HE) (*n* = 8). Mice allocated to the CD during the initial feeding period were also fed a CD during the intervention period (CC) (*n* = 8). This length of exposure to the exercise interventions has been previously shown to improve metabolic parameters in those males that undergo exercise interventions [17]. Animals were individually housed in a 12:12 h dark–light cycle for the entire study with ad libitum access to food and water. The use and care of all animals used in the study was approved by the Animal Ethics Committee of the University of Adelaide (Ethics approval code M-2011-093, approved Jan 2011) and were handled in accordance with the Australian Code of Practices for the Care and Use of Animals for Scientific Purposes.

### 2.2. Exercise Intervention (Swimming)

The swimming exercise regime was conducted as previously described [17].

### 2.3. Natural Mating to Produce Male Offspring

At intervention week 7 (21 weeks of age), founder males had the opportunity to mate with two normal-weight 10-week-old C57BL/6NHsd (Harlan) females for a maximum period of 8 days. Female mice were exposed to founder males during the dark cycle only and separated from the males and maintained on standard chow during the light cycle. Successful mating was assessed the following morning by the presence of a vaginal plug. After successful mating female mice were group housed until day 15 of pregnancy where they were individually housed until offspring were weaned. Mothers were maintained on standard chow during pregnancy and post birth. Mothers were allowed to pup and at weaning (day 21 of life) male offspring were separated from their mothers, group housed independently of founder treatment, and maintained on standard chow. For each independent measure of metabolic health one male was sampled per litter to reduce litter effects [23].

### 2.4. Body Composition

Pre-weaning body weights were recorded on post-natal days 5, 7, 10, 12, 14, and 21 with individual pups tracked. Post-weaning individual male offspring body weights were recorded up until 18 weeks of age. At 18 weeks of age, male offspring (*n* = 8 males per treatment), representing at least six founder males per treatment group, underwent a whole body composition of adiposity as measured by a dual-emission X-ray absorptiometry machine (DEXA) (Piximus, Ge Lunar, WI, USA) as previously described [24]. This was followed by a full post mortem where adipose depots (gonadal, omental, retro-peritoneal, peritoneal, and dorsal), liver, kidneys, and pancreas were collected and weighed; performed by the same individual blinded to founder treatment group.

### 2.5. Glucose and Insulin Tolerance

At 8 and 16 weeks of age a glucose tolerance test (GTT) was performed by intra-peritoneal (IP) injection of 2 g/kg of 25% d-glucose solution after 6 h of fasting. Insulin tolerance test (ITT) was performed at 9 and 17 weeks of age by IP injection of 0.75 IU of human insulin (Actapid^®^, Novo Nordisk, Bagsvaerd, Denmark) in a fed state. Tail blood glucose concentrations were measured using a glucometer (Hemocue, Angelholm, Sweden) at time points 0 (pre-bolus basal), 15, 30, 60, and 120 min. Data were expressed as mean blood glucose concentration per group as area under curve (AUC) for GTT and area above the curve (AAC) for ITT.

### 2.6. Male Offspring Insulin Secretion

At 16 weeks of age, male offspring (*n* = 7 males per treatment), representative of four founder males per treatment group, underwent insulin secretion during a GTT. At time points 0 (pre-bolus basal), 15, 30, and 60 min an additional 50 µL of blood was obtained via the tail vein using a Pasteur pipette. Whole blood was spun at 4000 rpm for 5 min and between 5 and 10 µL of plasma was removed and frozen at −20 °C until further testing. Insulin concentrations were determined by an Ultra-Sensitive Mouse Insulin ELISA Kit (#90080, Crystal Chem Inc., Downer Grove, IL, USA) as per manufacturer’s instructions. Data were expressed as mean whole blood insulin concentration per group as area under curve (AUC) and insulin secretion relative to the glucose stimulus at 0, 15, 30, and 60 min.

### 2.7. Metabolites and Hormone Analysis

At 18 weeks of age males were fasted overnight and blood plasma was collected at post mortem by a cardiac puncture under anaesthetic of 5% Avertin (2-2-2 Tribromethanol, Sigma-Aldrich, St. Louis, MO, USA). Plasma cholesterol, free fatty acids (FFAs), glucose, and triglycerides were measured on a Cobas Integra 400 plus automated sampler system (Roche, Basel, Switzerland) as per manufacturer’s instructions.

### 2.8. Male Offspring Pancreatic Histology and Islet Cell Morphology

At 8 weeks of age a subset of males (*n* = 6 males per treatment) that represented six founder males from CC, HH, and HE groups was assessed for pancreatic histology. The pancreas was cut in half. Half was snap frozen in liquid nitrogen and stored at −80 °C and the other half was fixed in 4% paraformaldehyde and stored in 70% ethanol. For sectioning, each pancreas was embedded in wax using standard methods and 7-µm sections were cut and heat fixed onto super frost slides. Each slide contained four 7-µm sections cut 50 µm apart. The sections were stained as previously described [25] to differentiate insulin producing β cells, scanned at 40× using the Nanozoomer Digital Pathology Scanner (Hamamatsu Co., Shizouka, Japan), and analysed using NDPview2.0 software (Hamamatsu Co.). The following parameters were measured: islet size, beta cell area (total beta cell area/total pancreas section area), and islet density (total number islets per 0.1 mm^2^ tissue). Islets were defined as packed clusters of ≥5 insulin-positive cells and were classified by size where 1 = <5000 µm^2^, 2 = 5000–10,000 µm^2^, and 3 = >10,000 µm^2^, as described previously [8]. Islet density was measured in 20 fields of view (0.1 mm^2^) per mouse.

### 2.9. Male Offspring Pancreas MicroRNA Analysis

Pancreatic tissues were homogenized using Precellys^®^ 24 (Bertin Technologies, Montigny le Bretonneux, France). Total pancreas RNA was extracted by standard TRI reagent extraction and isopropanol precipitation (Sigma-Aldrich, St. Louis, MO, USA). RNA concentration was quantitated with Qubit^®^ RNA HS Assay Kit on the Qubit^®^ 2.0 Fluorometer (Molecular Probes, Life Technologies, Carlsbad, CA, USA) whilst RNA integrity was assessed with Bioanalyzer (Agilent Technologies, Santa Clara, CA, USA). Prior to performing real-time quantitative polymerase chain reaction (qPCR) on microRNA (miRNA) arrays for pancreas miRNA expression profiling, miRNAs were reverse transcribed into cDNAs using Megaplex Rodent RT Primer Pool Set v3.0 (Applied Biosystems, Life Technologies, Foster City, CA, USA) followed by preamplification with Megaplex Rodent PreAmp Primer Pool Set v3.0 (Applied Biosystems). TaqMan^®^ Array Rodent MicroRNA A + B Cards Set v3.0 (Applied Biosystems) was used to interrogate the miRNA content of pancreas. *C*_t_ values were normalized to the mean *C*_t_ of the four probe sets for U6 small nuclear RNA (snRNA) and the fold change in miRNA expression between groups was determined by the ΔΔ*C*_t_ method. The Ingenuity® Pathway Analysis (IPA^®^) tool (Qiagen, Hilden, Germany) was used to generate a list of messenger RNA (mRNA) targets that were either experimentally confirmed to be direct targets of the miRNAs or predicted to be targets using a high-stringency filter. These targets were differentially expressed in the pancreas of male offspring born to HFD fathers and were restored to an abundance similar to that of offspring born to CD-fed males. A core network analysis was then performed using only experimentally observed interactions between molecules (i.e., strict setting) to predict the molecular networks that these mRNA targets are known to function in.

### 2.10. Statistics

All data were expressed as mean ± standard error mean (SEM) and checked for normality using a Kolmogorov–Smirnov test and equal variance using a Levene’s test. Statistical analysis was performed in SPSS (SPSS Version 18, SPSS Inc., Chicago, IL, USA) with AUC and AAC calculated in GraphPad Prism (GraphPad Software v6, San Diego, CA, USA). A *p* value < 0.05 was considered to be significant and the statistical analysis accepted if power of the model was ≥80%.

*Founder phenotype* was analysed by a general linear model with a least significant difference (LSD) post hoc test. In the model cohort of males a covariate was added to adjust for cohort variants.

*Offspring pre-weaning weights* were analysed using repeated measures ANOVA. In the model, father identification number (ID) and mother ID were included as a random effect to adjust for dependence in results between offspring from the same father and mother and litter size as a fixed variable to compare litter size variations between and within treatments.

*Adult offspring measures* were analysed using either a linear mixed-effect model or repeated measures ANOVA. In the model, father ID was included as a random effect to adjust for dependence in results between offspring from the same father, litter size was a fixed variable to compare litter size variations between and within treatments, and age was a fixed variable to determine if there were any effects due to age.

*Offspring pancreatic microRNA expression* fold change in miRNA expression between groups was determined by the ΔΔ*C*_t_ method and differences determined by a Student’s *t*-test on Δ*C*_t_ values (i.e., target microRNA *C*_t_ normalised to mean U6 *C*_t_).

## 3. Results

### 3.1. Founder Male Phenotype

The HFD founder phenotype was consistent with our previous studies that used this model of diet-induced obesity [12,17,18,26]. During the initial feeding period animals fed a HFD (which subsequently became HH and HE) gained significantly more weight as both an absolute value and percentage of weight gained compared with animals fed a CD (Table 1, *p* < 0.01). This increase in weight was primarily due to an increase in the percentage of adipose tissue (Table 1, *p* < 0.01). After the intervention period, both HH and HE males remained heavier than CC males (CC 29.3 ± 1.1 g, HH 35.8 ± 0.9 g, HE 32.3 ± 0.9 g, *p* < 0.05), however HE males were lighter than HH males (*p* = 0.05). This was because HH males continued to gain weight between 9 and 18 weeks, while HE males maintained the same weight as at the completion of the initial feeding period at 9 weeks (Table 1, *p* < 0.05). Again, the increased weight for HH and HE, compared with CC males, was primarily due to an increased percentage of adipose tissue (Table 1, *p* < 0.05), with HE males also having reduced adiposity compared with HH males (Table 1, *p* = 0.06). Exercise intervention (HE) in founder males improved glucose tolerance as measured by a AUC compared with HH males, restoring it to similar levels of CC males (Table 1, *p* < 0.05), however they remained insulin resistant, as measured by AAC compared with CC males (Table 1, *p* < 0.05). Fasting plasma cholesterol concentrations were higher in HH males compared with CC males (Table 1, *p* = 0.06), with HE males not different to either groups (Table 1).

### 3.2. Exercise Interventions in Fathers Partially Restore Pre-Weaning Weights in Male Offspring

**Figure 1:**
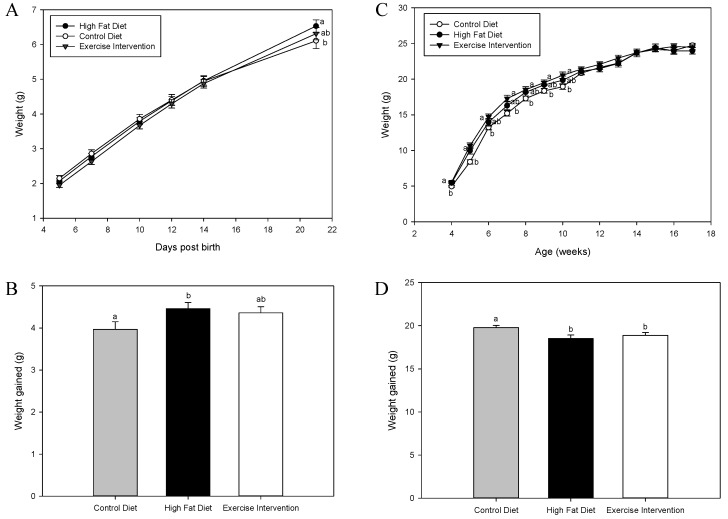
Effect of high fat diet fed founder diet and/or exercise interventions on male offspring pre-weaning and post-weaning weights. (**A**) Pre-weaning weights (day 5–day 21); (**B**) Total weight gained pre-weaning (day 5–day 21); (**C**) Post-weaning weights (4–17 weeks) and (**D**) total weight gained post-weaning (4–17 weeks). Data is expressed as mean ± standard error mean. ^a, b^ Different letters denote significance at *p* < 0.05.

There was no effect of founder diet and/or exercise intervention on pup weights from neonatal day 5 until post-natal day 14 (*p* > 0.05, Figure 1A). However, by post-natal day 21, male offspring born to HFD-fed fathers were heavier than male offspring born to fathers fed a CD (*p* < 0.05, Figure 1A), with male offspring born to fathers who under took exercise intervention not being different to either group (*p* > 0.05, Figure 1A). This was mirrored in total weight gained during the pre-weaning period, with male offspring born to HFD-fed fathers gaining more weight than male offspring born to CD-fed fathers (*p* < 0.05, Figure 1A). In addition, male offspring born to fathers that underwent exercise had an intermediate response – resulting in no difference to either group (*p* > 0.05, Figure 1B). Post-weaning, male offspring born to HFD-fed fathers remained heavier compared with male offspring born to CD-fed fathers until 11 weeks of age (*p* < 0.05, Figure 1C). Similarly, male offspring born to fathers that underwent exercise intervention were heavier compared with male offspring born to CD-fed fathers until 5 weeks of age (*p* < 0.05, Figure 1C), but were a similar weight to males born to HFD-fed fathers. By 6 weeks of age, their total body weight was not different to male offspring from either CC or HH groups (*p* > 0.05, Figure 1C). When assessing total weight gained during the post-weaning period (4–17 weeks of age), male offspring born to CD-fed fathers gained more weight compared with male offspring born to HFD-fed fathers or exercise intervention fathers (*p* < 0.05, Figure 1D), which were not different (*p* > 0.05, Figure 1D).

### 3.3. Exercise Intervention in Fathers Restores Glucose and Insulin Sensitivity in Male Offspring

**Figure 2:**
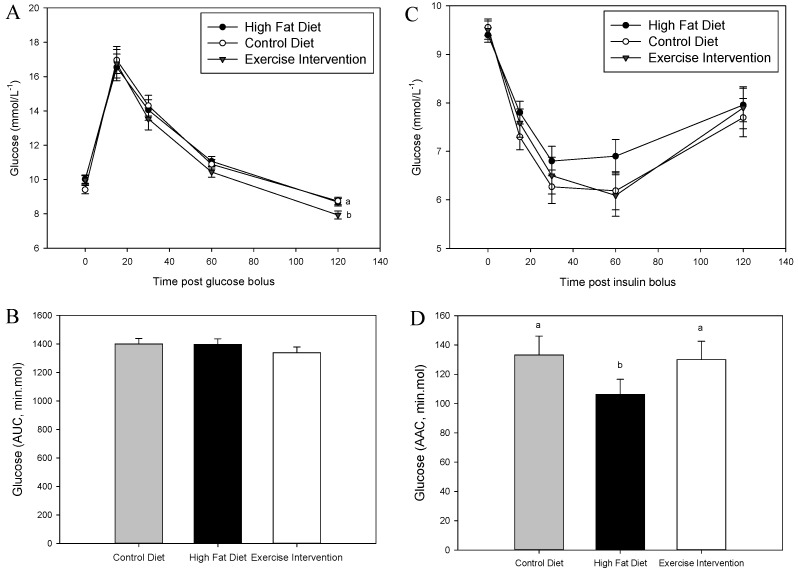
Effect of paternal high fat diet with exercise interventions on male offspring response to a glucose and insulin challenge (8–9 weeks). (**A**) Glucose tolerance as assessed by glucose tolerance test (GTT, 2 g/kg); (**B**) Glucose area under the curve (AUC, min·mmol) during GTT (**C**) Insulin tolerance as assessed by the insulin tolerance test (ITT, 0.75 IU) and (**D**) glucose area above the curve (AAC, min·mol) during ITT. Data is expressed as mean ± standard error mean. *n* = 10 male offspring from *n* = 10 litters representative of *n* = 8 fathers per group. ^a, b^ Different letters denote significance at *p* < 0.05.

**Figure 3:**
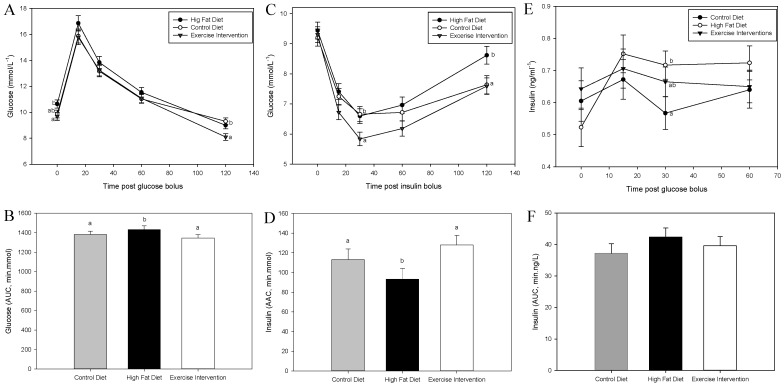
Effect of paternal high fat diet with exercise interventions on male offspring response to a glucose and insulin challenge (16–17 weeks). (**A**) Glucose tolerance as assessed by glucose tolerance test (GTT, 2g/kg); (**B**) Glucose area under the curve (AUC, min·mmol) during GTT; (**C**) Insulin tolerance as assessed by insulin tolerance test (ITT, 0.75 IU); (**D**) Glucose area above the curve (AAC, min·mol) during ITT; (**E**) Insulin secretion during a GTT and (**F**) insulin area under the curve (AUC, min·ng) during GTT. Data is expressed as mean ± standard error mean. *n* = 10 male offspring from *n* = 10 litters representative of *n* = 8 fathers per group. ^a, b^ Different letters denote significance at *p* < 0.05.

There was no effect of founder diet and/or exercise on male offspring glucose tolerance at 8 weeks of age (*p* > 0.05, Figure 2A,B). However, male offspring born to fathers fed an HFD had reduced insulin sensitivity at 9 weeks as indicated by a muted glucose response during an ITT and a reduced AAC compared with male offspring born to CD-fed fathers (*p* < 0.05, Figure 2C,D). By 16 weeks of age, male offspring born to HFD-fed fathers were glucose intolerant, as evidenced by an increased glucose response and a greater AUC during a GTT compared with male offspring born to CD-fed fathers (*p* < 0.05, Figure 3A,B). This glucose intolerance in male offspring from HFD-fed fathers was likely due their reduced insulin sensitivity, having a higher blood insulin concentration at 30 min during the GTT (*p* < 0.05, Figure 3E), a numerically higher insulin AUC during a GTT (Figure 3F) and reduced insulin tolerance during an ITT (*p* < 0.05, Figure 3C,D) compared with male offspring from CD-fed fathers. Interestingly, all of these negative impacts on glucose and insulin tolerance in male offspring from HFD-fed fathers were restored in offspring born to fathers that underwent the exercise intervention (*p* < 0.05, Figure 3) with insulin sensitivity further improved compared with male offspring of CD-fed fathers (*p* < 0.05, 30 min time point, Figure 3C).

### 3.4. Exercise Intervention in Fathers Reduced Total Adiposity and Plasma Lipids in Male Offspring

At 18 weeks of age, total adiposity both in absolute measures (grams) and as a percentage of total body weight was reduced in males born to fathers who underwent exercise intervention compared with both male offspring born to HFD- and CD-fed fathers (*p* < 0.05, Table 2), concomitant with an increase in percentage lean mass (*p* < 0.05, Table 1). There was no effect of father’s diet and/or exercise on bone density or total body weight in male offspring (*p* > 0.05, Table 2). Liver and kidney weights in both grams, percentage of total body weight, and pancreas weight as a percentage of total body weight were increased in male offspring born to HFD-fed fathers compared with male offspring born to CD-fed fathers (*p* < 0.05, Table 2). Again, exercise interventions in fathers restored these organ weights in their male offspring to that of the controls (Table 2). Furthermore, male offspring born to fathers that underwent exercise intervention had reduced soleus mass compared with offspring of the other two groups (*p* < 0.05, Table 2). There was no effect of founder diet and/or exercise on other organs and tissues measured (Table 2). Male offspring born to fathers fed a HFD had increased fasting plasma cholesterol compared with male offspring born to CD-fed fathers (*p* < 0.05), which was restored back to levels of offspring from CC-fed fathers if the father underwent exercise intervention (Table 2). In addition, male offspring born to a father that underwent exercise intervention had reduced FFAs compared with male offspring from both groups (*p* < 0.05, Table 2). There was no effect of founder diet and/or exercise on fasting plasma glucose, triglycerides, or insulin levels (Table 2).

### 3.5. Paternal Obesity Reduced Islet Cell Number and Size in Male Offspring, Which Was Partially Restored by Exercise Intervention in Fathers

**Figure 4:**
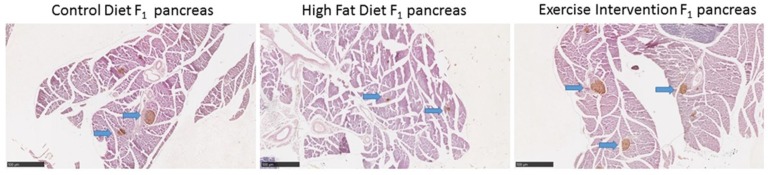
Effect of high fat diet fed founders with exercise interventions on male offspring pancreas morphology at 8 weeks. Representative pictures of pancreatic morphology and islet cell staining in male offspring. Images have been captured at 40x objective. Blue arrows are pointing to insulin positive cells (islet β cell clumps).

A known pancreatic phenotype associated with the development of metabolic syndrome is reduced islet cell numbers and size [27]. We therefore assessed pancreatic morphology in 8-week-old male offspring prior to any changes in glucose tolerance. Paternal HFD feeding reduced islet cell numbers and large islet cell size in male offspring compared with male offspring born to CD-fed fathers (*p* < 0.05, Table 3 and Figure 4). Exercise in the HFD-fed father restored islet cell numbers in male offspring to those of male offspring born to CD-fed fathers (Table 3 and Figure 4). There was no effect of founder diet and/or exercise on male offspring pancreas weight (g and % of body weight), the proportion of islet cell size categories, or percentage of β-cell area (*p* > 0.05, Table 3 and Figure 4).

### 3.6. Paternal Obesity Alters Pancreatic MicroRNA Expression inMale Offspring, Which Was Partially Restored by Exercise Intervention in Fathers

**Figure 5:**
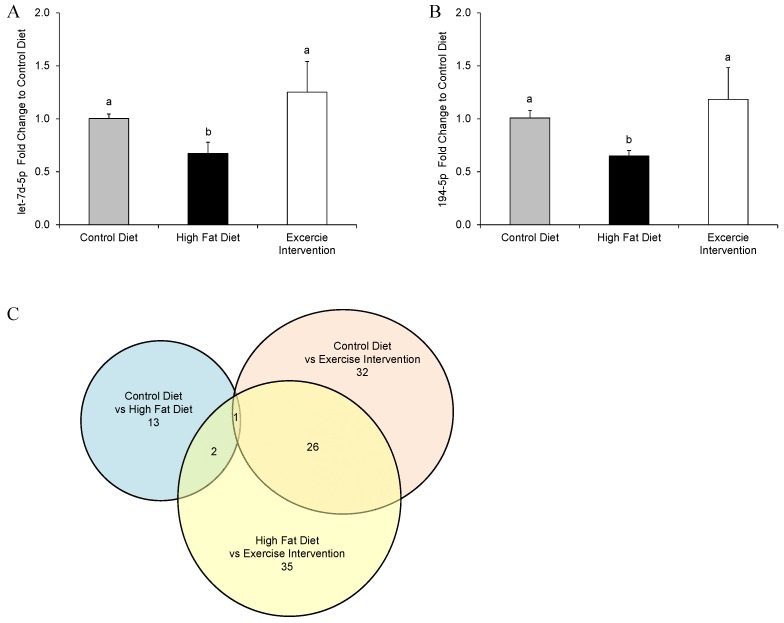
The effect of a paternal high fat diet with exercise interventions on the expression of pancreatic microRNAs in male offspring. The expression of microRNA (**A**) let7d-5p and (**B**) 194-5p in the pancreases of male offspring was reduced by a paternal HFD and restored by an exercise intervention to an abundance that was not different to that of offspring sired by CC-fed fathers; (**C**) the number of differentially expressed microRNAs between groups and the number of differential microRNAs in common between groups. Data is presented as mean fold change ± standard error mean by ΔΔ*C*_t_ (**A**,**B**) or number of microRNAs (**C**). *n* = 4 male offspring; representative of *n* = 4 fathers per group. ^a, b^ Different letters denote significant difference at *p* < 0.05.

MicroRNAs have been shown to be important in regulating gene expression networks involved in organ development, cellular growth/proliferation, and cell death, which may in turn impair pancreas function and lead to the impairment of glucose and insulin homeostasis. As such, microRNAs are currently being investigated as potential biomarkers for type II diabetes [28] and metabolic syndrome [29]. Therefore, we assessed the expression of 641 murine microRNAs in the pancreases of male offspring by a TaqMan^®^ microRNA rodent array cards to determine if the expression of any microRNAs were restored by paternal exercise interventions. Of the 13 pancreatic microRNAs with differential expression between male offspring born to HFD-fed fathers compared with CD-fed fathers, two were restored by the paternal exercise intervention (microRNAs let7d-5p and 194-5p; Figure 5A,B) to an abundance similar to that of animals born to CD-fed fathers. One microRNA (microRNA 190a-5p) was differentially expressed in all groups (down-regulated by a paternal HFD; up-regulated by the paternal exercise intervention—compared with a paternal CD). It must also be noted that a greater number of male offspring pancreatic microRNAs were differentially expressed due to the paternal exercise intervention (32 microRNAs) and between the paternal exercise intervention group and the paternal HFD group (35 microRNAs); with many of the same microRNAs dysregulated (26 microRNA; Figure 5C). Interestingly, none of the altered 13 microRNAs in pancreatic tissue of male offspring born to HFD-fed fathers were the same of those altered in sperm of founder males fed this same HFD [13].

Ingenuity pathway analysis limited to targets of microRNAs let7d-5p and 194-5p that were either experimentally observed or predicted using a high stringency filter were used to build networks with experimentally-observed connections between network molecules. The functions that the top five pathways are involved in include cell growth, cell proliferation, cell death, gene expression, organ morphology, and RNA post-transcriptional modification (Table 4).

## 4. Discussion

An ever-growing number of animal models have demonstrated that male obesity at conception can program health pathologies in offspring [6,7,8,9,10,11]. However, very few studies have determined whether this transgenerational programming of offspring health by paternal obesity can be reversed through interventions aimed at the father. We have previously shown that diet and/or exercise interventions in obese fathers prior to conception restored insulin sensitivity and adipose accumulation in female offspring [12]. Surprisingly, the intervention of low to moderate exercise, without a change in the founder’s high-fat diet, resulted in the greatest improvements to female offspring metabolic health, thus indicating that the positive benefits of the exercise regime overrode the negative effects of the consumption of a high-fat diet. We therefore extended this hypothesis to include the impacts of this low to moderate exercise program in obese fathers, without a diet change, on the metabolic health of their male offspring. We demonstrated that exercise interventions in high-fat diet-fed fathers is capable of somewhat normalising the metabolic profiles of male offspring similar to those of male offspring born to CD-fed fathers. Interestingly, this paternal exercise intervention also further reduced gross adiposity, circulating FFAs, and modified pancreatic microRNA abundance in offspring beyond that of control animals.

### 4.1. Prolonged Paternal HFD Feeding Induces Glucose Intolerance, Insulin Resistance, Pancreatic Islet Cell Dysfunction, and Hyperlipidaemia in Male Offspring

Similar to previous studies [7,10], paternal obesity was induced by HFD feeding prior to conception and resulted in altered pre-weaning and post-weaning growth, glucose intolerance, insulin resistance, and hyperlipidaemia of male offspring. Here, we further demonstrate that male offspring born to HFD founders also have reduced pancreatic islet cell numbers, reduced islet cell size, and dysregulation of pancreatic microRNAs—all prior to the onset of glucose intolerance. It must be noted that some discord remains in the literature surrounding as to whether pancreatic histological changes are associated with the development of metabolic syndrome and type II diabetes. However, human studies indicate that β-cell mass is decreased in type II diabetes as well as in hyperinsulinemia and that the underlying mechanism is increased cellular apoptosis [30,31]. Therefore, similar histological changes to pancreatic tissue in our male offspring born to fathers fed a high-fat diet may be occurring, predisposing them to later impaired glucose tolerance. These observations for male offspring are consistent with the histological and molecular changes seen in pancreatic tissue of female offspring born to fathers fed a high-fat diet [8,9] and likely also explains their accumulation of metabolic syndrome with age. 

### 4.2. A Short-Term Founder Exercise Intervention Normalised the Metabolic Profile of Male Offspring

Male offspring born to fathers who underwent exercise intervention displayed normalisation of glucose tolerance, insulin sensitivity, cholesterol concentrations, pancreatic morphology, and microRNA abundance to the same levels are those of offspring born to CD-fed fathers. This mirrors our findings in female offspring born to fathers who underwent this same exercise intervention [12]. Interestingly, while male offspring born to fathers who underwent exercise had insulin tolerance and FFA concentrations similar to those of their sisters, differences in body composition and glucose tolerance were evident between the sexes. For instance, male offspring had reduced total adiposity compared to offspring born to both control and fathers fed a high-fat diet, while only changes to adipose cell size and adipose accumulation were seen in the corresponding group of female offspring. These differences may result from the dissimilar growth trajectories observed between male and female offspring. For example, female offspring had increased weight gain between 8 and 16 weeks, whilst male offspring had reduced weight gain seen during this same time period compared with their contemporary controls. Additionally, glucose tolerance was not different between the groups of female offspring, whilst glucose tolerance was different in male offspring born to HFD-fed fathers at 16 weeks of age with complete restoration in male offspring born to fathers that underwent exercise. Overall, this suggests that as a result of paternal programming, male offspring are more prone to glucose intolerance in early life than female offspring, who were able to adapt by increasing insulin production. These sex-specific differences in phenotypes are consistently observed as a result of paternal programming and are most likely related to sex-specific changes emanating from early embryonic development [7,8,32,33]. 

The results presented in this paper indicate that the exercise program in the founder males counteracted any negative effects of high-fat diet consumption (founder insulin resistance, and/or increased adipose accumulation), as evidenced by offspring born to males undergoing the exercise intervention not inheriting insulin resistance or increased adiposity. Indeed investigations in human cohorts have reported that single short-term bouts of exercise, without a change to diet or with limited change to adiposity, improved glucose tolerance due to adaptive changes in skeletal muscle metabolism [34]. Similar to the human findings our founder males that underwent exercise interventions had improved glucose tolerance, similar to males fed a CD, despite maintaining increased adiposity. This in agreement with our earlier reports that in a rodent model of obesity paternal glucose tolerance predicts both sperm function and offspring metabolic health, independent of founder adiposity [12,17]. Additionally, paternal blood glucose concentrations can predict offspring serum glucose levels in both male and female offspring [35]. Taken together, this suggests that the positive effects of exercise on founder glucose metabolism could be one of the main mediators of the improvements observed in our offspring, identifying an additional marker that could be used to assess the effectiveness of other intervention strategies than exercise.

While offspring metabolic health was nominally restored at 18 weeks of age by the paternal exercise intervention, a closer inspection of their pancreatic tissue prior to the onset glucose intolerance (in males from HFD fathers), revealed only partial restoration of islet cell area and size. In addition, at this same age only two out of the 13 dysregulated microRNAs in male offspring pancreatic tissue from HFD fathers were restored back to expression levels of control offspring (let7d-5p and 194-5p). However, these two microRNAs that were restored target mRNAs that act in molecular networks that function in key pathways involved in cell growth, cell proliferation, cell death, gene expression, organ morphology, and RNA post-transcriptional modification. Disruption of target pathways by these microRNAs may have led to the observed metabolic phenotypes of male offspring born to HFD-fed fathers, and the partial rescue of this phenotype in offspring born to fathers that underwent exercise. Indeed, it has been previously demonstrated that the dysregulation of let7 in the pancreas of mice led to glucose intolerance [36]. This suggests that interventions that improve paternal metabolic health also hold the potential to, at least partially, normalise molecular signals in offspring pancreatic tissue.

### 4.3. Exercise Intervention in Founders Further Changed Adipose Accumulation, Circulating FFAs, and Pancreatic MicroRNA Abundance in Male Offspring

One of the most interesting findings from this study was with respect to the differences in offspring phenotypes from our control group and our exercise intervention group. Male offspring born to HFD-fed fathers that underwent exercise interventions had reduced gross adipose tissue and circulating plasma FFAs, as well as altered pancreatic microRNA abundance compared with male offspring born to CD-fed fathers. This suggests that not only is paternal exercise capable of rescuing offspring phenotype, but that the restoration of metabolic phenotypes may not result from the same molecular processes as those of the offspring of CD-fed fathers. It has previously been reported that women who exercise prior to conception and during gestation have reduced chances of foetal macrosomia, irrespective of body mass index prior to commencing the exercise regime [37]. Similarly, pre-conception exercise in fathers reduced adipose tissue and increased muscle mass in male offspring. These are important markers of offspring health as increased adipose accumulation and decreased muscle mass pre-empt metabolic syndrome [38]. Therefore, increased muscle mass in our male offspring born to fathers that underwent exercise interventions might explain their improved glucose and insulin metabolism. Furthermore, circulating free fatty acids and lipids also predict the development of metabolic syndrome in men [39]. Given that offspring of fathers that underwent exercise interventions also displayed reduced plasma FFAs and cholesterol, this too potentially influenced their improved glucose and insulin tolerance.

Perhaps the changes in offspring metabolic profile that were enhanced beyond those of control animals due to founder exercise might also explain the greater number of differentially expressed pancreatic microRNAs. This again highlights that the molecular pathways triggered to restore the metabolic health in these offspring may differ to those of offspring from CD fathers. How exercise in fathers maintained on a HFD alters these offspring phenotypes compared with those of controls remains to be elucidated, however, differentially abundant X-linked microRNAs in founder sperm have previously been implicated to enact this phenomenon in female offspring [12]. In humans, short bouts of exercise can alter the microRNA profile of serum [40,41], therefore a similar mechanism could also be acting here, as sperm-borne microRNAs are known to program offspring phenotypes [14,42]. Sperm-derived small non-coding RNAs have been clearly implicated in the paternal transmission of metabolic syndrome to offspring; this was highlighted by recent publications implicating not only sperm-borne microRNAs [14,43] but also transfer RNA (tsRNA) (the most abundant RNA in sperm) [15,44] and tsRNA modifications [45,46]. Given that the injection of sperm RNAs of 30–40 nt in length (predominantly tsRNAs) into zygotes recapitulated the offspring metabolic phenotype caused by a paternal high-fat diet [15], it is also likely that paternal exercise also modulates sperm tsRNA abundance. Further investigations are warranted to determine the range and magnitude of this potential alteration. 

In contrast to our observed beneficial effects on offspring phenotypes due to exercise in obese male mice is a study published by Murashov et al. [47]. They subjected male C57BL6 mice to 12 weeks of voluntary wheel running whilst being fed standard chow prior to mating, which resulted in founders with reduced adiposity but unaltered glucose and insulin homeostasis. Offspring born to fathers that underwent this intense exercise program were heavier, had increased fat accumulation, were glucose intolerant, and had higher fasting insulin levels, indicating metabolic syndrome. These findings conflict with our results that exercise in obese fathers restored metabolic profiles of offspring to match those of controls. Divergent study designs and founder phenotypes are the likely reason for these discrepancies. Firstly, we used mice that had been subjected to a HFD challenge for 9 weeks and therefore prior to the exercise intervention our mice had increased adiposity and altered lipid profiles. Secondly, our exercise program modelled light to moderate intensity for a period of 9 weeks, which included an acclimatisation phase to minimise stress of the obese animals entering into the exercise program. This is particularly important as paternal stress by itself can initiate metabolic disturbances in offspring; Thirdly, our exercise regime resulted in a maintenance of body weight and improved glucose tolerance, which was not observed by Murashov et al. [47], indicating that the systemic effects of weight loss from relatively intense forms of exercise might be detrimental to sperm quality. However, differences between these studies highlight the importance of tailoring exercise programs to individual circumstances, so that any intervention designed for obese males can maximise health benefits to offspring phenotypes.

## 5. Conclusions

This study demonstrates that low to moderate exercise interventions in obese fathers prior to conception not only improves circulating metabolic parameters whilst maintaining adiposity in founders, but also provides a targeted and simple intervention strategy to reduce the effect of obesity-induced programming of impaired metabolic health in the next generation.

## Figures and Tables

**Table 1:** Effect of founder high-fat diet (HFD) with exercise interventions on body composition and metabolites.

	Control Diet	High-Fat Diet	Exercise Intervention
**Pre intervention**			
Total body weight (g)	26.6 ± 1.3 ^a^	32.6 ± 1.1 ^b^	32.1 ± 1.1 ^b^
% of weight gained	28.0 ^a^	43.0 ^b^	45.0 ^b^
Adipose tissue (% of total body weight)	15.1 ^a^	25.3 ^b^	24.7 ^b^
**Post intervention**			
Total body weight (g)	29.3 ± 1.1 ^a^	35.8 ± 0.9 ^b^	32.3 ± 0.9 ^c,^*
% of weight gained	9.2 ^a^	11.2 ^a^	0.2 ^b^
Adipose tissue (% of total body weight)	17.5 ^a^	28.2 ^b^	23.7 ^c,&^
**Metabolites**			
Glucose AUC (min·mmol)	1670 ± 131 ^a^	2106 ± 110 ^b^	1562 ± 111 ^a^
Insulin AAC (min·mmol)	148 ± 17 ^a^	102 ± 14 ^b^	93 ± 15 ^b^
Cholesterol (mmol/L^−1^)	3.14 ± 0.49 ^a^	4.39 ± 0.42 ^b,#^	3.90 ± 0.41 ^a,b^

Data is expressed as mean ± SEM. Data represents *n* = 6 founders per treatment. ^a, b, c^ Different superscript letter denote significance at *p* < 0.05. * Exercise intervention (HE) different to high-fat diet (HH) at *p* = 0.05. ^&^ HE different to HH at *p* = 0.06. ^#^ HH different to control diet (CC) at *p* = 0.06. AAC: area above the curve; AUC: area under curve.

**Table 2:** Effect of founder high fat diet with exercise interventions on male offspring body composition and metabolites (18 weeks old).

	Control Diet	High-Fat Diet	Exercise Intervention
Total body weight	24.9 ± 0.3	25.4 ± 0.3	24.3 ± 0.3
**DEXA Body Composition**			
**Grams (g)**			
Adipose tissue	2.07 ± 0.06 ^a^	2.07 ± 0.06 ^a^	1.76 ± 0.06 ^b^
Lean Mass	22.0 ± 0.3	22.1 ± 0.3	21.6 ± 0.3
Bone	0.43 ± 0.01	0.42 ± 0.01	0.44 ± 0.01
***% of total body weight***			
Adipose tissue	8.31 ± 0.20 ^a^	8.17 ± 0.21 ^a^	7.29 ± 0.21 ^b^
Lean Mass	88.1 ± 0.6 ^a,b^	86.9 ± 0.7 ^a^	89.0 ± 0.7 ^b^
Bone	3.77 ± 0.13	3.54 ± 0.14	3.84 ± 0.15
**Post Mortem Body Composition**			
**Grams (g)**			
Liver	0.87 ± 0.02 ^a^	1.04 ± 0.02 ^b^	0.92 ± 0.02 ^a^
Pancreas	0.15 ± 0.01	0.17 ± 0.01	0.15 ± 0.01
Kidneys	0.27 ± 0.01 ^a^	0.29 ± 0.01 ^b^	0.25 ± 0.08 ^a^
Soleus (mg)	8.44 ± 0.48 ^a^	9.70 ± 0.47 ^a^	6.43 ± 0.47 ^b^
Vastus Lateralis (mg)	144.9 ± 5.7	159.7 ± 5.7 ^a,b^	145.0 ± 4.5
**% of total body weight**			
Gonadal Adiposity	1.08 ± 0.09	1.04 ± 0.09	0.98 ± 0.09
Omental Adiposity	0.20 ± 0.03	0.23 ± 0.03	0.16 ± 0.03
Perirenal Adiposity	0.09 ± 0.02	0.09 ± 0.01	0.08 ± 0.01
Retro Adiposity	0.14 ± 0.02	0.12 ± 0.02	0.10 ± 0.01
Dorsal Adiposity	0.39 ± 0.02	0.39 ± 0.02	0.39 ± 0.02
Total sum Adiposity	1.89 ± 0.14	1.86 ± 0.14	1.71 ± 0.12
Liver	3.67 ± 0.10 ^a^	3.98 ± 0.10 ^b^	3.84 ± 0.10 ^a,b^
Pancreas	0.61 ± 0.02 ^a^	0.67 ± 0.02 ^b^	0.63 ± 0.02 ^a^
Kidneys	1.13 ± 0.02 ^a^	1.11 ± 0.02 ^a,b^	1.06 ± 0.02 ^b^
**Metabolites**			
Glucose (mmol/L^−1^)	9.15 ± 0.60	7.97 ± 0.69	8.54 ± 0.66
Cholesterol (mmol/L^−1^)	2.31 ± 0.01 ^a^	2.41 ± 0.02 ^b^	2.32 ± 0.01 ^a^
FFA (mmol/L^−1^)	0.43 ± 0.01 ^a^	0.42 ± 0.01 ^a^	0.39 ± 0.01 ^b^
Triglycerides (mmol/L^−1^)	0.51 ± 0.03	0.42 ± 0.03	0.40 ± 0.03
Insulin (ng/L^−1^)	0.61 ± 0.06	0.52 ± 0.06	0.64 ± 0.07

Data is expressed as mean ± standard error mean *n* = 10 male offspring from *n* = 10 litters representative of *n* = 8 fathers per group. ^a, b^ Different superscript letters denote significance at *p* < 0.05. DEXA: dual-emission X-ray absorptiometry machine; FFA: free fatty acid.

**Table 3:** Effect of founder high fat diet with exercise interventions on male offspring pancreas morphology at 8 weeks.

	Control Diet	High-Fat Diet	Exercise Intervention
Pancreas (g)	0.115 ± 0.010	0.123 ± 0.008	0.116 ± 0.009
Pancreas (% of body weight)	0.44 ± 0.03	0.52 ± 0.03	0.51 ± 0.03
Islet cell density (0.1 mm^2^)	0.42 ± 0.07 ^a^	0.29 ± 0.06 ^b^	0.35 ± 0.07 ^a,b^
Small islets (%) (0–5000 µm^2^)	62	65	68
Medium islets (%) (5000–10,000 µm^2^)	20	15	12
Large islets (%) (>10000 µm^2^)	18	20	20
Small islet size (µm^2^)	1864 ± 578	1900 ± 491	2180 ± 506
Medium islet size (µm^2^)	6771 ± 1124	6551 ± 827	7446 ± 1111
Large islet size (µm^2^)	20,983 ± 930 ^a^	15,930 ± 1200 ^b^	17,778 ± 945 ^a^
β-cell area (%)	0.43 ± 0.11	0.32 ± 0.09	0.37 ± 0.10

Data is expressed as mean ± standard error mean *n* = 6 male offspring from *n* = 6 fathers and *n* = 6 litters per group. ^a, b^ Different superscript letters denote significance at *p* < 0.05.

**Table 4:** The top five ranked molecular networks identified by Ingenuity pathway analysis of microRNA targets.

#	Molecules in Network	IPA Score	Focus Molecules	Top Diseases and Functions
1	**ARHGAP5, ATG10, BICD2, CALU, CARHSP1** *, **CEP164, COL14A1, CSNK2A1, CTPS1, DNAJA2, ESPL1, FKBP6, FLRT2, HDLBP, IGDCC4, KMT2C, KPNA1, NRARP, PMEPA1, PPFIBP1, PPP2R2A, PTPRU, RAP2B, RBM38, REV3L, RTKN, SEMA6A, SH3BGRL2, SMARCAD1, SPCS2** *, **TMEM43, TP53, UBR5, VAPA, ZMAT3**	38	35	Cancer, Cell Cycle, Hematological Disease
2	**BBX, CHD4, CHD7, DNMT3A, DOT1L, DUSP7, DZIP1, E2F5, E2F6, EZH2, FBXO32, FGF5, GCNT4, GLI3,** HISTONE H3, HOXA9, IDH2, IKBKAP, LOR, MEIS2, MYCN, RAB38, RAG1, RBBP4, SALL1, SMARCC1, SOX17, SS18, ST3GAL1, STK40, TLR4 *, TOR1AIP2, UHR *, USP12, ZIC1	36	34	Gene Expression, Cell Death and Survival, Embryonic Development
3	**AGO3, AKT2, ANKRD49, ANO1, ATP6V1G1, C5ORF51, CAP1, CPD, GLIS3, HLF, HNB, ID4, MTMR2, MYRF, NR2F2, NUDT4, P2RX1, PHLDA1, PIGA, PLEKHH1, PPARA, PPP1R16B**, PRO-INSULIN, RFFL, SECISBP2L, SLC22A23, SNX30, SOCS7, SYPL1 *, TCF7L2, TGFßR1 *, TMEM2 *, TPP1, UCHL5, UGT8 *	36	34	Digestive System Development and Function, Endocrine System Development and Function, Organ Morphology
4	ACSL6, ACVR1C, ARID3B, ATP6V1H, CSNK1D *, DICER1 *,EGR3, EIF3J *, ELK4, ERCC6, HAS2, HIC2, HS3ST2, IGDCC3, IGF2BP2 *, KLF8, LPIN2, MAP2, NOTCH, NRIP1, PAG1, PBX3, PLXND1, POLR2C *, PSME3, RARA, RICTOR, RNAPOL II, **RORC, SCN11A**, SECRETASE Γ, SLC16A10, SLC25A24 *, SYT4, TARBP2	31	32	RNA Post-Transcriptional Modification, Cellular Development, Cellular Growth and Proliferation
5	ACTA1, ACTIN, ALP, ARHGAP20, BMP2K *, CAPG *, CAPN3, CSRNP3, DMD, FZD3, FZD5, GAN, GATM, GPCPD1, GSK3, IGR, KCNC1, LDB3, LHX1, LHX6, MAP4K3, OTP, SEC14L1, SLC25A13, SLC45A4, SLC46A3, SNAP91, TET2, TMEM108, TRIB2, TTC39C, TTC7B, TTL, XK, ZFHX3	31	32	Cellular Development, Cellular Growth and Proliferation, Embryonic Development

Pathway analysis was limited to experimentally observed interactions between molecules and experimentally observed/predicted (high stringency) mRNA targets. Focus molecules with * are experimentally observed targets of microRNAs let-7d-5p and 194-5p, whereas bold molecules predicted (high stringency) targets. RNA = Ribonucleic Acid. IPA = Ingenuity® Pathway Analysis.
